# Remarks on Multimodality: Grammatical Interactions in the Parallel Architecture

**DOI:** 10.3389/frai.2021.778060

**Published:** 2022-01-04

**Authors:** Neil Cohn, Joost Schilperoord

**Affiliations:** Department of Communication and Cognition, Tilburg School of Humanities and Digital Sciences, Tilburg University, Tilburg, Netherlands

**Keywords:** multimodality, linguistic theory, parallel architecture, grammar, codeswitching

## Abstract

Language is typically embedded in multimodal communication, yet models of linguistic competence do not often incorporate this complexity. Meanwhile, speech, gesture, and/or pictures are each considered as indivisible components of multimodal messages. Here, we argue that multimodality should not be characterized by whole interacting behaviors, but by interactions of similar substructures which permeate across expressive behaviors. These structures comprise a unified architecture and align within Jackendoff's Parallel Architecture: a modality, meaning, and grammar. Because this tripartite architecture persists across modalities, interactions can manifest within each of these substructures. Interactions between modalities alone create correspondences in time (ex. speech with gesture) or space (ex. writing with pictures) of the sensory signals, while multimodal meaning-making balances how modalities carry “semantic weight” for the gist of the whole expression. Here we focus primarily on interactions between grammars, which contrast across two variables: symmetry, related to the complexity of the grammars, and allocation, related to the relative independence of interacting grammars. While independent allocations keep grammars separate, substitutive allocation inserts expressions from one grammar into those of another. We show that substitution operates in interactions between all three natural modalities (vocal, bodily, graphic), and also in unimodal contexts within and between languages, as in codeswitching. Altogether, we argue that unimodal and multimodal expressions arise as emergent interactive states from a unified cognitive architecture, heralding a reconsideration of the “language faculty” itself.

## Introduction

Natural human communication combines speech, bodily movements, and drawings into multimodal messages (McNeill, 1992; Goldin-Meadow, 2003a; Kress, 2009; Bateman, 2014; Bateman et al., 2017). Rarely does speech or writing appear in isolation, but rather we gesture when we talk and we combine pictures with text. Yet, models of language competence typically do not account for this diversity of expression. Consider the well-known phrase “I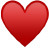 NY,” created by designer Milton Glaser, as seen on t-shirts, mugs, posters, and other paraphernalia. When seen for the first time, one has to connect the heart image to the linguistic construction [_S_ Subject—Verb—Object] in order to recognize that the heart is playing a role as a verb, specifically to mean LOVE. Now consider the following sentences, all taken from real-world contexts:

(1)

a) I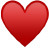 making new friends (Twitter post)
b) Please drive slowly. We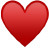 our children. (Street sign)
c) They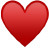 weddings (Twitter post)
d) I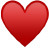 transitive pictograph verbalizations (T-shirt)

Across these examples, the heart plays a role in the uninflected verb position carrying the consistent meaning (and possibly pronunciation) of LOVE. Repeated exposure to these kinds of expressions may lead one to generalize the heart in different sentences while playing this role, which overall creates a construction in the form of [_S_ Subject—
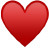
_V_–Object], a pattern even self-referentially appearing in (1d). Thus, the heart—a graphic sign—has become a part of the written English lexicon.

**Figure 1:**
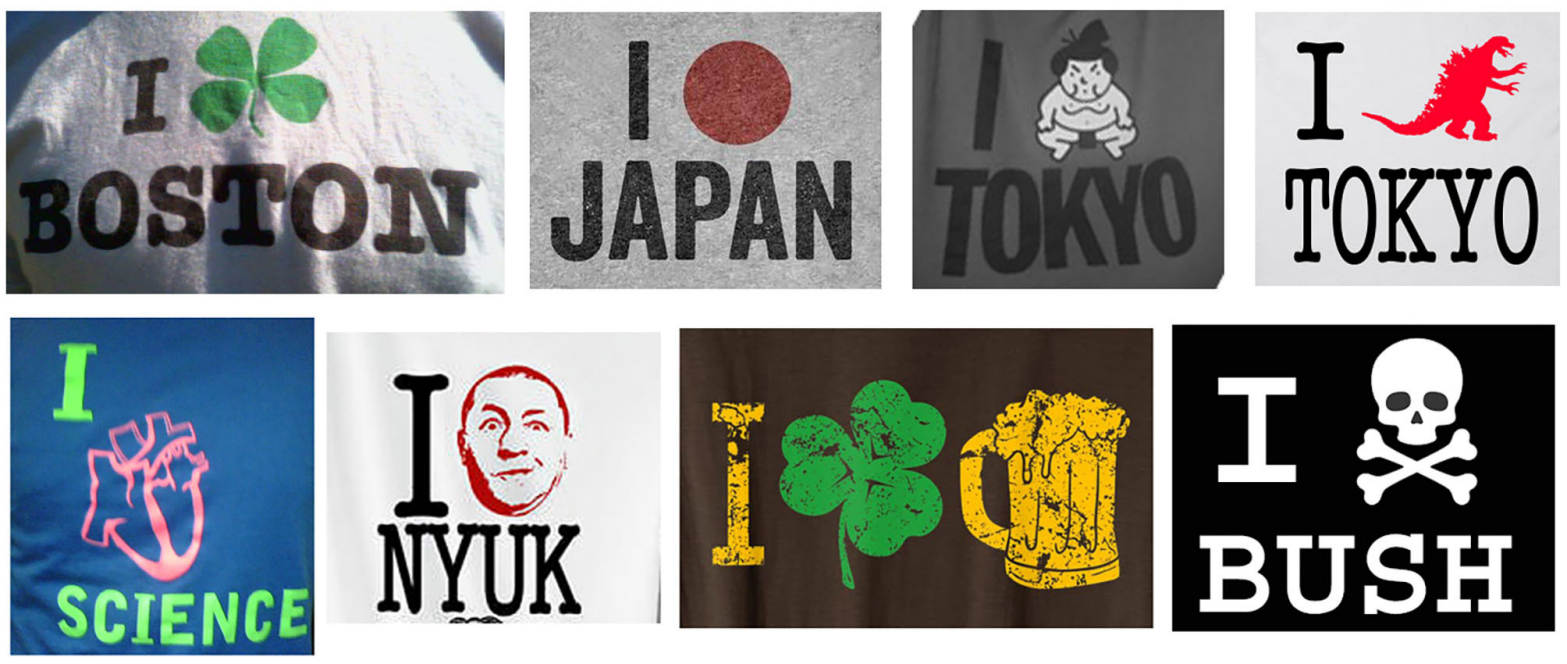
T-shirts all using a pattern of Subject—Picture_Verb_–Object.

Now consider the sentences in Figure 1, all from t-shirts, which each use a picture in the verb position, but which do not carry as deterministic meaning or pronunciation as the heart. Rather, the semantics of the pictures-as-verbs either maintain the meaning of “love” and/or invoke semantic relatedness to the Direct Object of the sentence. Following the original construction for New York, the pattern may involve an Object that is a place, but with a verb-picture related to that place, like for Tokyo with a sport played there (sumo) or a monster that destroys it (Godzilla). However, this pattern can be used beyond places. For example, “Nyuk” is an utterance typically made by Curly from The Three Stooges, whose face appears in the verb position of that sentence, while the skull-and-crossbones comes from an activist t-shirt reflecting a displeasure with a former U.S. president.

These examples imply a further, more general construction of [_S_ Subject—Picture_Verb_–Object] where the verb slot of the canonical sentence structure (N-V-N) must be filled by a picture, not a written word, that semantically connects to the Direct Object. This construction is more general than the heart-construction, since the open verb can be filled by any image associated with the Direct Object, not just a heart. This forms an abstract grammatical pattern, but with slots mandatorily filled by different modalities.

These patterns form a taxonomy of entrenched relationships from general (S-Picture_V_-O) to constrained (S-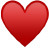
_V_-O) to specific (I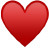 NY). People learn these patterns from encountering instances repeatedly, which then allow for abstraction to a generalized construction. Crucially, these are multimodal patterns, which all leave their traces in our lexicons, but involve more modalities than just speech/writing. In fact, many stored multimodal patterns involve both fixed patterns and constructional variables. Productive schemas have been identified in multimodal memes shared on social media (Dancygier and Vandelanotte, 2017; Schilperoord and Cohn, 2022), and emoji which are systematically integrated with written language across digital communication (Gawne and McCulloch, 2019; Weissman, 2019). In addition, gestures have long been recognized as integrated with speech in ways that question their separability (McNeill, 1992; Goldin-Meadow, 2003a), and indeed have been argued to have constructional properties (Lanwer, 2017; Ladewig, 2020).

Because these multimodal patterns entwine forms of spoken and written language with those of other modalities, accounting for these phenomena requires discussing them in terms of the language system. In fact, we contend that any *complete* theory of language must account for how elements from other modalities are integrated with the verbal lexicon. We may thus articulate these issues as fundamental questions involved in a theory of “knowledge of language,” expanding on those articulated by Jackendoff and Audring (2020) for unimodal language:

1. What elements does a speaker/signer/drawer store in memory, and in what form?
2. How are these elements combined online to create novel (multimodal) utterances?
3. How are these elements acquired?

As demonstrated by the picture-substitution constructions described above, and attested by decades of research on co-speech gesture, multimodal expressions that involve the body or graphics cannot be separated from the linguistic system. Such interactions are not between “language” and other “external” systems, given that encoded lexical items themselves may integrate multiple modalities. Such integration heralds a single system—an architecture of language—that covers such multimodal expressions in full. We contend that in order to accurately characterize the natural manifestation of language, multimodality must be incorporated into the cognitive model of language.

In fact, such an architecture is already available in Jackendoff's Parallel Architecture (Jackendoff, 2002; Jackendoff and Audring, 2020), and a first attempt at extending it as a multimodal model was taken in Cohn (2016). We here clarify, refine, and elaborate on this approach. In the sections below we first provide an overview of our multimodal expansion of the Parallel Architecture. We then focus specifically on questions exemplified by our examples above: how do grammatical structures interact across and within modalities?

## Multimodal Parallel Architecture

Many theories characterize multimodal expressions as built of indivisible “modalities” — such as speech, gesture, or pictures—which then interact (Fricke, 2013; Bateman et al., 2017; Forceville, 2020). However, most linguistic models agree that language is composed of interacting mental structures—phonology, syntax, semantics—that give rise to a holistic experience of speech. Thus, to describe the holistic experience of a multimodal expression, we aim to first identify the mental structures that comprise those expressions, and then describe how these structures are interacting. These structures are not found in the culturally manifested expressions “out there” in the world, but instead in the minds of people that construct and comprehend those expressions. Thus, ingredients of language itself—and of multimodal interactions—are not the “features” or “characteristics” that one can describe about the messages, but rather are the mental structures that coalesce to allow those expressions.

**Figure 2:**
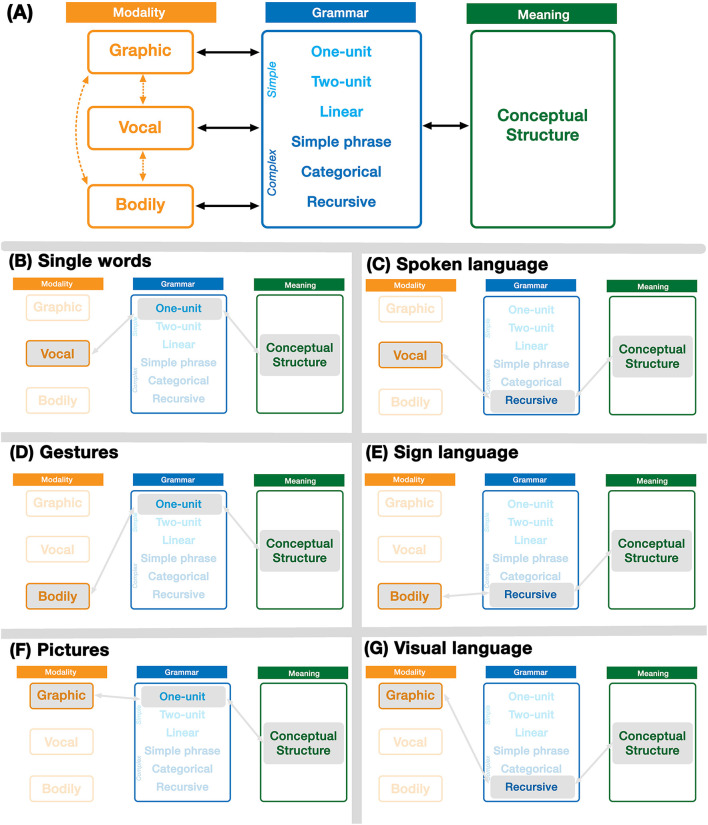
The Parallel Architecture including multiple modalities. Expressive forms arise through emergent states within the full architecture **(A)**. These include single unit expressions **(B,D,F)** and potentially full languages using recursive grammars **(C,E,G)**.

In line with the mental structures described for language—phonology, semantics, and syntax—but abstracted, these three components are: Modality, Meaning, and Grammar. Following Jackendoff's Parallel Architecture (Jackendoff, 2002; Jackendoff and Audring, 2020), each of these components are mutually interfacing. In addition, each structure allows for combinatoriality using the operation of Unification, a principle of assembling schematic structures. We describe each of these structures in brief below, with the full architecture presented in Figure 2A.

### Modality

A modality is the channel by which a message is expressed and conveyed. We take a modality to involve a cluster of substructures that include the sensory apparatus for producing and comprehending a signal, the cognitive structures that guide such signals, and the combinatorial principles that govern how those signals combine. For example, speech uses the vocal modality, which is produced through oral articulation, and perceived via the auditory system. It uses phonemics to codify signals, which are combined using phonological structures. Gesture and sign language would use the bodily modality, which is produced through articulation of the body (hands, torso, face, etc.) in different positions and movements, perceived through the visual and/or haptic system. Bodily signals are instantiated cognitively as the bodily equivalent of phonemics and phonology, which appear to be different than those guiding speech (Pa et al., 2008). Finally, pictures manifest in the graphic modality, which is produced through bodily motions that leave traces to make marks, which are perceived through the visual system. These marks are decoded as graphemes, guided by combinatorial structures of a “graphology” (Willats, 1997; Cohn, 2012).

While each natural modality is optimized for particular types of messaging, cross-modal correspondences between modalities have also emerged. For example, *writing* maps the natural vocal modality (speech) into the natural graphic modality (drawing) to create an unnatural correspondence for graphic depictions of speech, which repurposes neural areas naturally associated with the visual system (Hervais-Adelman et al., 2019). Sign languages have also attempted to be graphically instantiated in their own writing systems (Sutton, 1995), and indeed various gestures appear in graphic form in the emoji vocabulary (Gawne and McCulloch, 2019), not to mention gesticulations drawn in pictures more widely (Fein and Kasher, 1996). In Figure 2A, we notate these cross-modal mappings between modalities with dotted lines.

Thus, the vocal, bodily, and graphic modalities constitute the three natural modalities that humans have available to express conceptual structures. In the original Parallel Architecture, only phonology addressed modalities, which was largely characterized in terms of the vocal auditory modality. While the “phonology” of sign language was alluded to, it was left ambiguous as to whether “phonology” in the Parallel Architecture was conceived as a modality-specific construct (i.e., the auditory-vocal modality) or whether it was a modality-general construct with different sensory manifestations (i.e., “phonology” could serve as the broader class for all modalities). The multimodal Parallel Architecture makes it explicit, that all modalities are present *at once*, as depicted in Figure 2A. The important implication is that “language” is not an amodal representation that “flows out” of different modalities, but rather all modalities are present and persisting as part of a larger holistic communicative faculty, whether or not expressions in those modalities rise to the level of full languages (as in Figures 2B–G, and discussed further below).

### Meaning

A second component of language and communicative systems is their capacity to convey meaning. We follow Jackendoff (1983, 1987, 2002) in calling this Conceptual Structure, a modality-sensitive “hub” of semantic memory which aggregates semantic information from across sensory and cognitive systems (Jackendoff, 1987; Kutas and Federmeier, 2011; Ralph et al., 2016). Conceptual structure is fundamentally combinatorial and constituting an independent level of structure, using intrinsically semantic units, like objects, paths, events, properties, and quantifiers. The specific ways that these structures may manifest depends on the modality, i.e., speech and graphics convey meaning in different ways, or on the representational systems within a modality, i.e., English and Swahili differ in how they convey meaning.

We here follow Jackendoff's (1983; 1987; 2002) model of Conceptual Semantics in articulating these conceptual structures, which we believe provides formalisms which can best express multimodal semantic relationships in explicit terms, including the emergent inferences that multimodality may evoke. We should note that the full treatment of meaning in the Parallel Architecture also includes a Spatial Structure which articulates an abstract geometric representation of meaning. In our full treatment of multimodal semantics in works to come, we include both systems of Conceptual Structure and Spatial Structure, but for simplicity we here omit Spatial Structure.

### Grammar

The final component of languages is that of Grammar, the system that packages meaning in order to be expressed. While taxonomies of grammars have been posited for the vocal and bodily modalities (Chomsky, 1956; Jackendoff and Wittenberg, 2014), recent work has argued for comparable architectural principles to operate in the sequencing of graphics, particularly in visual narratives like comics (Cohn, 2013c). Neurocognitive research has found similar neural responses to manipulations of picture sequences as those observed in sentence processing (Cohn, 2020b), consistent with findings of shared resources for verbal syntax and music (Patel, 2003; Koelsch, 2011). Indeed, human neurocognition has been posited as allowing for a range of combinatorial sequencing (Dehaene et al., 2015), which could thus manifest in different representations across modalities.

We here make a broad distinction between the complexity of types of grammatical expressions (Jackendoff and Wittenberg, 2014, 2017; Dehaene et al., 2015). ***Simple*** ***grammars*** contribute little to the organizational structure of a sequence beyond the information provided from conceptual structures. They package information as a single unit, two-units, or a linear sequence, whereby the meaning of the units alone motivates organization of the utterance. In contrast, ***complex grammars*** contribute structure to the message they organize, by assigning categorical roles to differentiate units and by segmenting sequences into constituents, possibly with recursive embedding. Because basic memory capacity is fairly limited for sequencing meaningful information on its own, representations of distinguishable types (categorical grammar) and segmentation (simple phrase grammar) are posited to facilitate more complex sequencing, and thus more complex meaningful expressions. We elaborate further on types of grammars below.

### Unimodal Expressions

We contend that full languages instantiate the three components of Modality, Meaning, and Grammar in a balanced way. Jackendoff's (2002) Parallel Architecture accounted for these interactions for the vocal modality to describe the structures of spoken languages, and alluded to the bodily modality to describe sign languages. Our extension of the Parallel Architecture thus includes all three natural human modalities of the vocal, bodily, and graphic structures which persist in parallel within a single unified system. All expressive modalities then arise out of emergent interactions between these component parts of the Parallel Architecture. Thus, spoken languages involve an interaction of the vocal modality with a complex grammar and conceptual structures (Figure 2C). Sign languages involve a similar emergent interaction with the bodily modality (Figure 2E), again along with a complex grammar and conceptual structures.

Because the structures in the Parallel Architecture are independent yet mutually interfacing, these same components can yield expressions that may lack certain structures, not fully manifesting as languages with all three components (Cohn, 2013b). For example, expressions of the modality alone in the vocal modality would yield non-sense vocables like *sha-la-la-la-la* or non-words like *fwiggle* and *plord*. In the bodily modality this would be non-meaningful bodily expressions, and in the graphic modality this would yield non-meaningful mark-making as found in abstract art.

We draw primary attention here to meaningful expressions that lack a complex grammar, remaining as single unit expressions or as unstructured linear sequences. For example, vocal expressions using simple grammars guided by their meaning alone include single unit expressions such as *ouch, pow*, or *kablooey*, which do not have grammatical categories allowing them to be combined into well-formed sentences (Jackendoff, 2002; Jackendoff and Wittenberg, 2014), as illustrated in Figure 2B. Similarly, gestures in the bodily modality are typically single expressions (Figure 2D) lacking a complex grammar, particularly emblems like *thumbs up* or gestural expletives, which may appear in isolation. When produced multimodally, gestures appear at a rate of once per spoken clause (McNeill, 1992; Goldin-Meadow, 2003a). These single bodily motions may also differ in the degree to which they are instantiated in memory, whether as novel gesticulations, entrenched emblems, or gestural constructions (McNeill, 1992; Goldin-Meadow, 2003a; Ladewig, 2020). In all cases, these simple expressions lack the complex grammars that characterize spoken and sign languages.

While most research has focused on the vocal and bodily modalities, we further argue that these components also extend to the graphic modality. Single unit meaningful graphic expressions are many pictures (Figure 2F), which might range in internal complexity from depicting full scenes (such as drawings and paintings) to simpler signs (such as emoji or pictograms used in signage). More recent work has argued that sequential drawings in visual narrative sequences use a narrative grammar with categorical roles and recursive constituent structures (Cohn, 2013b,c), and manipulation of this structure evokes similar neural responses as the syntax of sentences (Cohn, 2020b). Because these graphic systems again use all three components of a modality (graphics), meaning, and grammar (narrative), we argue that this constitutes a *language* in the graphic form as well: ***visual language*** (Cohn, 2013b).

Thus, expressions across the vocal, bodily, and graphic modalities use a combination of a Modality, Meaning, and Grammar. Crucially, when those correspondences between a piece of Modality, Grammar and Meaning get fixed, they constitute lexical items, i.e., stored representations encoded in the interfaces between levels of structure (Jackendoff and Audring, 2020). That is, we maintain that the lexicon is distributed across all structures of the Parallel Architecture (Modality, Grammar, Meaning), for lexical items of varying sizes and complexity, and such breakdown dissolves the boundaries between lexicon and grammar (i.e., because grammatical schemas are stored within and across the lexicon). This addresses our first question above, about what elements are stored in memory. The multimodal Parallel Architecture which we argue for here thus predicts that lexical items can appear in all modalities, including for the bodily modality in the lexicons of sign languages and gestural emblems, and in the extensive visual lexicons of drawings and graphic representations (Forceville, 2011, 2019; Cohn, 2012, 2013b; Schilperoord and Cohn, 2021).

**Figure 3:**
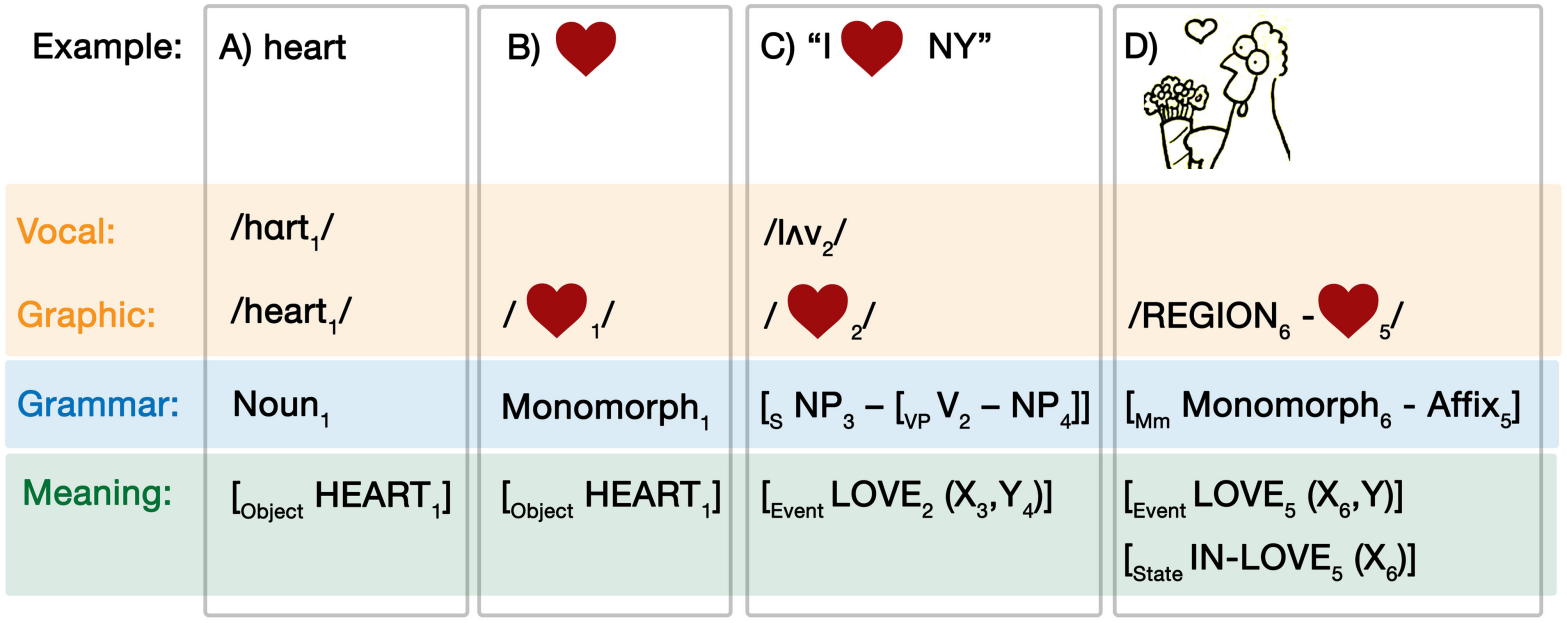
Lexical entries for **(A)** the word “heart,” **(B)** the heart shape, **(C)** a multimodal construction using the heart shape, and **(D)** the heart shape as a visual affix.

Consider the vocal word “heart,” for which we provide the lexical entry in Figure 3. As a spoken word, it has a three-part structure of its modality, phonology: /hɑrt/, graphic spelling: /heart/, its grammar as a noun, and its meaning as an object HEART. The correspondences across levels of structure are marked by subscripted indices, here “1.”

We can similarly specify a lexical encoding for the heart-shape, which in fact has multiple entries. First, a simple heart-shape in Figure 3B has the modality of its pictorial form, which is grammatically a *monomorph*—a isolatable visual form that can stand alone (Cohn, 2018; Schilperoord and Cohn, 2021)—while its conceptual specification is also the object HEART. Because the word “heart” shares its meaning with and provides a label for the heart-shape, the word (Figure 3A) and base image (Figure 3B) can be thought of as sister schemas (Jackendoff and Audring, 2020). This is expressed by the similar indices (“1”) shared across levels and modalities in (2a) and (2b).

The heart-shape has at least two additional entries. First, as discussed in our introduction, it can be used as a verb in a construction, as in the sentence “I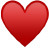 NY.” The heart-shape within this construction is characterized in Figure 3C. Here, the heart-shape is pronounced with the phonology/lʌv/and plays the role of a verb in a canonical sentence schema, which has the conceptual structure of an event of LOVE with arguments corresponding to the noun phrases. This example shows how not only words are encoded in a lexicon, but also grammatical constructions with open slots that can be filled by other encoded lexical items. It also shows that lexical items can be multimodal, i.e., encoded across modalities.

An additional encoding of the heart-shape concerns its usage as a visual affix in graphic representations, as in Figure 3D, such as an “upfix,” an object that floats “up” above a character's head to indicate a cognitive or emotional state (Cohn, 2013b, 2018). The graphic form of this usage places a heart-shape above or near a character's head, shorthanded here to “REGION” for a visual region of an image that would act as a visual variable. At the level of the visual grammar, the heart-shape corresponds to an affix, which cannot stand alone, attaching to the character which is a monomorph to then form a larger monomorph (Cohn, 2018). This corresponds to two potential meanings: a transitive case when the heart reflects the event LOVE with arguments for its morphological stem and some other entity (i.e., the chicken loves something), or, alternatively, a state of an argument corresponding to the morphological stem as being IN-LOVE (i.e., the chicken is in love).

Expressions in any modality thus make use of, and can combine, these encoded lexical items which include information from all three components of the Parallel Architecture. Stored lexical items can range in size from pieces of form-meaning mappings (like affixes), to whole isolable forms (words, monomorphs) and to grammatical constructions. Because the range of complexity is accessible to all modalities, the combination of modalities within the model allows for multimodal constructions (as in Figure 3C).

As described above, our model posits that all modalities persist within the Parallel Architecture simultaneously, making use of semantics and grammar with modality-specific affordances. There is no “flow” of an “amodal” language into one modality or another (aside from cross-modal correspondences like writing), because all modalities are co-present and functional as part of a holistic system. We thus posit that the determination of a system's complexity depends on how it may become nurtured across development. The correspondences between each natural modality (vocal, bodily, graphic) and conceptual structures persist as “resilient” features (Goldin-Meadow, 2003b) of an innate, “core” meaning-making system. That is, humans innately have a capacity to create simple expressions (single units, linear sequences) of sounds, bodily motions, and drawings, which persist no matter the additional development. Modalities can further develop as full linguistic systems when also engaging substantial grammars and lexicons.

Thus, a person will develop a sign language if they receive the requisite exposure and practice with a system that provides them with a lexicon and grammar. Yet, even if a person does not learn a sign language, in typically-developing circumstances they retain their resilient ability to express meaning with gestures (Goldin-Meadow, 2003b), just as fluent signers also retain the use of gestures (Marschark, 1994; Emmorey, 2001). Similarly, if a person does not learn a full visual language (often reflected in the statement of “I can't draw”), they retain the ability to create basic drawings (Cohn, 2012). The complexity that each modality may develop into is thus determined by exposure to a representational system in one's environment. Nevertheless, no matter what level of complexity is achieved in development for each modality, all modalities persist as part of a holistic expressive system. These issues address the third question above about acquisition.

### Multimodal Expressions

**Figure 4:**
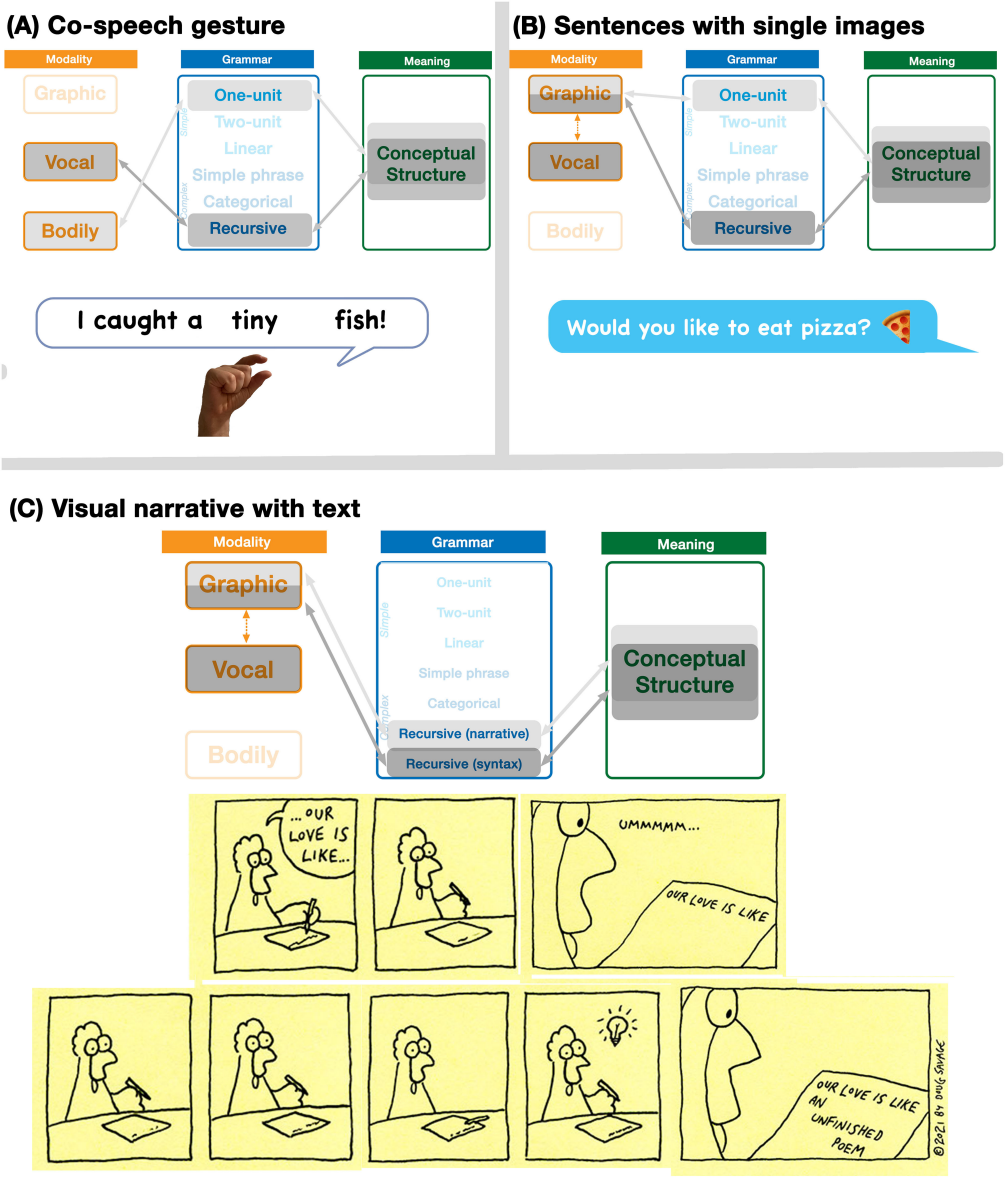
Multimodal interactions arising in the Parallel Architecture. Emergent states here describe **(A)** co-speech gesture, **(B)** text-emoji relationships, and **(C)** a visual sequence using a narrative grammar alongside grammatical text. *Savage Chickens* is © 2021 Doug Savage.

Since unimodal expressions across modalities can arise out of different activation patterns within the Parallel Architecture, multimodal expressions involve simultaneous emergent interactions of unimodal expressions. While there are numerous such potential emergent interactions, we provide three here as examples. First, consider someone saying the sentence *I caught a tiny fish* while simultaneously making a small pinching gesture. As diagrammed in Figure 4A, this interaction would involve a grammatically complex sentence with a one-unit gesture. The modalities and grammars remain independent of each other, but they correspond to a common conceptual structure, reflecting their shared and/or constructed meaning. Such convergence into a common conceptual structure aligns with McNeill (1992) notion of a “growth point,” the common origin of meaning across both speech and gesture.

Next, consider the interaction which occurs in Figure 4B, a combination of a written sentence and an emoji. Like in co-speech gestures, this interaction involves a grammatically complex sentence with another modality using a one-unit grammar, here an emoji instead of a gesture (Cohn et al., 2019; Gawne and McCulloch, 2019). Unlike the speech-gesture combination though, both expressions here originate from the graphic modality. What differs is the cognitive representation of these expressions. The pizza emoji is a natural visual representation, here represented by the light gray highlighting in the “graphic” modality box, along with light gray highlighting across grammar and meaning. The text is signaled here with the dark gray highlighting. Here, the vocal modality interfaces with the graphic modality to create the cross-modal correspondence reflected in writing (see the vertical doubly pointed arrow), which then subsequently interfaces with grammar and meaning.

Finally, consider the comic strip in Figure 4C. This representation again involves both pictorial and textual information in a shared graphic modality. However, both text and images use complex grammars. The written text uses syntactic structure (both uttered in, and “inherent” to, the world of the pictures), while the visual sequence involves a narrative structure which also uses recursive constituent structures (detailed further below, and in Figure 4). While only this complex interaction is diagrammed here, the sequence also involves a simple grammar in the one-unit utterance “ummmmm…” in the third panel. This sequence therefore uses two complex grammars in addition to the simple grammar, compared to the speech/gesture and text/emoji interactions which used a combination of complex and simple grammars.

## Relationships of Modality and Meaning

Within the Parallel Architecture, emergent interactions can give rise to unimodal and multimodal expressions. Given that this tripartite structure operates across all modalities of expression, each substructure internally has the possibility of interactions when mixing representations across or within modalities. That is, multimodality can involve interactions between modalities, between meanings expressed by modalities, and/or between the grammatical structures manifested in modalities. Interactions within each structure operate independently of the other structures, but coalesce in the broader expressivity of communication. For example, though all multimodal interactions in Figure 4 involve two modalities, the interface between speech and gestures differs in nature from the interface between writing and pictures. Similarly, the multimodal interactions between grammars in Figures 4A,B combine a complex grammar with a simple single unit, while the example in Figure 4C brings together complex grammars. These differences imply characterizable interactions between elements within each substructure of the Parallel Architecture. We here briefly discuss interactions between modalities and meanings, before progressing to more detail about grammars and their interactions.

### Modality Relations

Interactions between modalities are the primary way that we experience multimodality, since the sensory signals of modalities (light, sound) provide our only overt access to these messages (Jackendoff, 2007). When these sensory signals allow for a singular experience, we follow Clark (1996) in characterizing them as ***composite signals***. Composite signals are aggregations of multiple modalities together into a unified multimodal unit. This creation of unitized composite signals may not be a binary matter, but instead operates across a continuum. We here focus on two primary ways that composite signals may be made based on the affordances of modalities' sensory signals.

A first way that modalities interact is through the coordination of the modalities themselves. These relations can be characterized as *alignment* or *correspondence* between the sensory signals in different modalities (Rasenberg et al., 2020), which is constrained by the affordances of how different modalities convey information. For example, speech is produced vocally and received auditorily, while bodily motions are produced through the body and received visually (or haptically), yet both unfurl across a duration of time allowing their alignment. In such temporal correspondence, expressions may come with various degrees of synchrony to create a composite unit. Thus, a small pinching gesture depicting size might be predicted to align with the word “tiny” in *I caught a tiny fish*, not with the word “caught.”

Temporal correspondence between modalities can use the simultaneous production of modalities in time as a way to cue their relationship between each other. While the process of writing or drawing also unfurls in time (Willats, 2005; Cohn, 2012; Wilkins, 2016), this temporality often disappears once the process is completed, after which only a static form persists. Without duration to align modalities, relationships between pictures and words therefore use a spatial correspondence, through the degree to which modalities share a common region and/or use cues to integrate them into a composite multimodal unit (Cohn, 2013a). The least integrated type of multimodal interaction keeps text and pictures fully separate (as in most academic articles, including this one), while greater integration can be facilitated by devices like labels or speech balloons. For example, Figure 4C uses the phrase (*Our love is like…*) in two ways: it is interfaced to the images with the device of a speech balloon in the first panel, while the same phrase appears written on a piece of paper within the storyworld in panel 3. The text is the same in both cases, but it interfaces in two different ways with the pictures.

### Meaning Relations

Most theories of multimodality focus on categorizing the ways that modalities meaningfully interact (see Bateman, 2014 for review). These categorizations often expand on balanced or imbalanced semantic relationships where information expressed in one modality may support, elaborate, or extend the information expressed in another modality (Martinec and Salway, 2005; Royce, 2007; Kress, 2009; Painter et al., 2012; Bateman, 2014). We here characterize the global “balance” of meaning between modalities as the ***semantic weight*** of a multimodal utterance. When meaning is conveyed in one modality more than another, it carries more of the “weight” of the overall message. Below we characterize semantic weight as a binary distinction, but it is likely proportional along a scale (again in line with our “weight” metaphor).

Multimodal interactions that are ***balanced*** in their semantic weight involve multiple modalities with relatively equal contribution of meaning, while ***imbalanced*** semantic weight places the locus of meaning primarily in one modality. Consider the utterances in (2) as if they were sent as text messages:

(2)

a) Would you like to eat pizza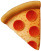 (imbalanced)

b) Would you like to eat pizza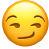 (balanced)

Both of these messages would be diagrammed as in Figure 4B, as sentences with a single emoji. In (2a) the sentence is followed by a pizza emoji, which is coreferential to the word “pizza” in the text. Deleting the pizza emoji would have little impact on the overall gist, suggesting that the writing is more informative and thus carries more semantic weight. Omission of the sentence however, leaving only the pizza, would certainly impact the meaning of the message. With the writing carrying more semantic weight than the image, it implies an imbalanced relationship.

This differs from (2b) where the winking face emoji implies an innuendo or at least some added information not conveyed by the text. Omission of either the winking emoji or the text here would alter the overall expression's gist, implying that both modalities substantially contribute, and thus have a balanced relationship. This balanced semantic weight arises in part because the smirking emoji here maintains no direct coreference to the units of the sentence, unlike the coreferential relationship between the word *pizza* and the pizza emoji in (2a). With no direct coreference, this multimodal relationship would then require further inferencing to resolve in the Conceptual Structures.

## Grammatical Complexity

Before progressing to describe relations between grammars, we first will elaborate on our broad categorization of grammars as either simple or complex by detailing the range of grammatical complexity, following Jackendoff and Wittenberg's (2014) hierarchy of grammars. In contrast to the idealization of grammatical structures in the classic Chomskyan hierarchy (Chomsky, 1956), this hierarchy provides a more ecological characterization of the complexity of combinatorial principles used to map form to meaning.

Jackendoff and Wittenberg's (2014) hierarchy of grammars is shown in Table 1, together with their basic schemas. As will be demonstrated, this hierarchy can be applied to characterize the sequencing across all modalities, and thus we have modified the terminology to apply to this broader context. In this sense, the hierarchy of grammars can be viewed as a modality-independent capacity, and the manifestation of grammars in different modalities may vary in the representations that they use. Although a spoken word and a graphic picture obviously differ in how they convey meaning, both represent a single isolable *utterance*, and thus we argue both are characterizable by the types of grammar in the hierarchy. Similarly, the syntactic structures used in verbal languages and the narrative structures used in visual languages differ in their representations, but both employ the same combinatorial principles. The hierarchy of grammars thus characterizes the abstract means of combinatoriality, which may become manifest in the representational schemas encoded in memory for a given system.

**Table 1:** A hierarchy of grammars by Jackendoff and Wittenberg (2014), with modified terminology to apply across modalities.

**Grammar complexity**	**Grammar type**	**Schemas**
Simple	One-unit grammar	[_Utterance_ Unit]
	Two-unit grammar	[_Utterance_ Unit—Unit]
	Linear grammar	[_Utterance_ Unit—Unit*]
Complex	Phrase structure grammar	[_Utterance_ Unit/Phrase*] [_Phrase_ Unit—Unit] (2-unit phrase) [_Phrase_ Unit*] (unlimited phrase)
	Categorical grammar	[_Utterance_ Unit_x_ –Unit${}_{\text{y}}^{*}$]
	Recursive grammar	[_Utterance_ Unit/Phrase*] [_Phrase_ Unit/Phrase*]

As argued above, simple grammars characterize organizations where conceptual structures motivate the sequencing, with little contribution from the grammar itself. At the most simple, ***one-unit grammars*** of verbal expressions include words like *abracadabra, gadzooks, ouch!*, or *ummmmm…* as in the third panel of Figure 4C, which are not encoded as syntactic categories to place them into sentence structures (Jackendoff, 2002; Jackendoff and Wittenberg, 2014). Ideophones, like *pow* or *kablam* also use one-unit grammars, generally maintaining morphosyntactic independence from their sentence contexts (Dingemanse, 2017). In the bodily modality, most gesticulations and gestural emblems remain as one-unit grammars that cannot be put into a coherent sequence (McNeill, 1992; Goldin-Meadow, 2003a), and individual pictures constitute single unit expressions in graphic form, whether complex compositions of whole scenes, like paintings, or simple units, like icons or emoji.

***Two-unit grammars*** are only slightly more complex. In speech, two-unit sequences appear in children's two-word stage of language learning and in pivot grammars (*Lake X* vs. *X Lake*) (Jackendoff and Wittenberg, 2014). Two-unit grammars can also characterize compounds, which allow for a wide range of meaningful relations between units (Jackendoff, 2009). In the visual modality, two-unit grammars are used across several constructions illustrating yes/no contrasts (as in signs showing which foods one can and cannot have in a classroom), or pairs of images denoting comparisons or before-after causal relations (Plug et al., 2018; Schilperoord and Cohn, 2022). Overall two-unit grammars allow a wide range of construals of relations between juxtaposed units which are not grammatically encoded.

Simple grammars without constraints on length may manifest as linear sequences with only meaningful associations, a ***linear grammar***. In speech this occurs in contexts like lists, the speech of some aphasics, and in languages that require only semantic heuristics to guide their sequencing (Jackendoff and Wittenberg, 2014, 2017). Visual sequences use these linear relations in visual lists, like in instructions about what to or not to carry onto a plane, what you can do in a park, or what tools to use when assembling furniture (Cohn, 2020a). Visual linear grammars also appear when people type numerous related emoji in an unstructured way, such as several emoji related to birthday parties (Cohn et al., 2019), as in: 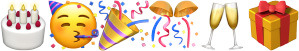.

In contrast to simple grammars, complex grammars contribute representational structure to their constituent parts beyond only semantic relations. ***Simple phrase grammars*** segment a sequence into constituent parts with one level of embedding. ***Categorical grammars***, called “part-of-speech grammars” by Jackendoff and Wittenberg (2014), differentiate the units in a sequence with roles which may function with varying salience and distributions in a sequence. In spoken and signed modalities, such categories are typically nouns, verbs, and other syntactic classes. Visual information seems to be less optimized for expressing sentence level parts-of-speech (Cohn et al., 2019), and manifest more naturally as narrative level categories (Cohn, 2013c, 2020b). Though we list simple phrase grammars and categorical grammars sequentially, they lie as various options at the same level of complexity.

**Figure 5:**
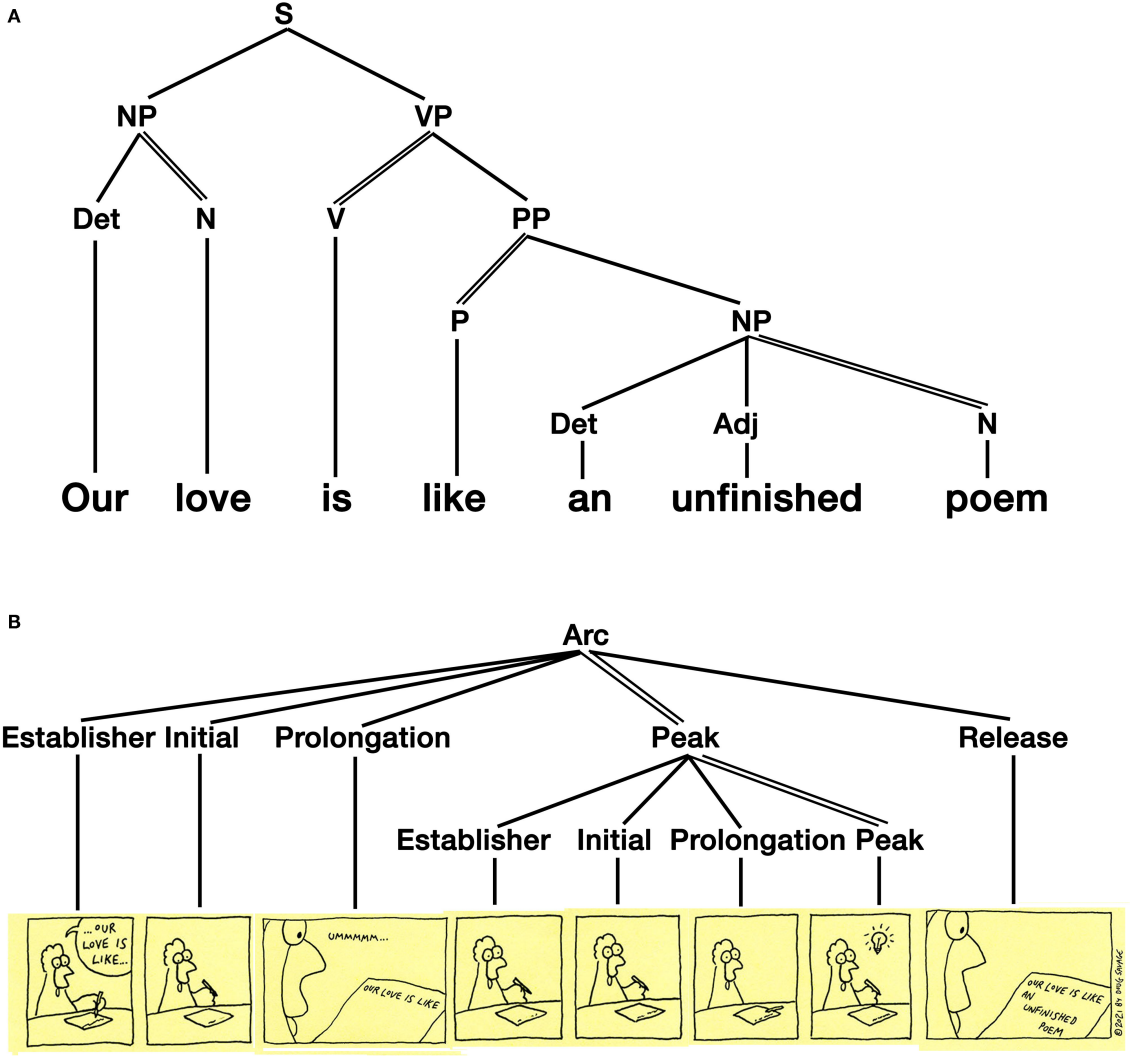
Grammatical structures in both **(A)** the syntactic structure of spoken languages and **(B)** as the narrative structures of visual languages. *Savage Chickens* is © 2021 Doug Savage.

Finally, ***Recursive grammars*** allow for the embedding of units or constituents of one type to embed in constituents of that same type. In Table 1, we use the notation of Unit/Phrase to indicate an concatenation of either units or phrases with a Kleene star that indicates that either units or phrases can extend to unlimited length, following the notation of Jackendoff and Wittenberg (2014). Recursive grammars are the most complex level of grammar and can manifest sentence structures, whether in spoken or sign language, as in Figure 5A. Recursive grammars have also been shown to organize narrative structures in visual sequences (Cohn, 2020b) which involves roles played by different units and recursive structures organizing units into hierarchic constituents. For example, the sequence in Figure 5B uses a canonical narrative schema (Establisher-Initial-Prolongation-Peak-Release) embedded within another narrative structure.

## Grammatical Relationships

Multimodal (and unimodal) interactions may involve expressions using grammars at various levels of the hierarchy of grammatical types. We now turn to detailing how these grammars might interact. Here we posit two principles that characterize the ways that grammars of different expressions and various complexity interact.

The first principle of grammatical interactions is ***symmetry***. Following the hierarchy of grammars, grammars broadly fall into classes of simple and complex types. Simple grammars provide little structure of their own, and merely facilitate a mapping between a modality and meaning without additional representations. Complex grammars further contribute representations of categorical roles and/or constituent structure. Given these two broad possibilities, we can characterize interactions between grammars as either *symmetrical* between grammars of the same type, i.e., simple with simple, or complex with complex, or *asymmetrical*, i.e., simple with complex. In the examples in Figures 4A,B, the full sentences (complex) interact with single-unit expressions (simple), thus creating asymmetrical interactions, while the use of two recursive relationships in Figure 4C exemplifies a symmetrical interaction.

The second principle concerns the ***allocation*** of grammars relative to each other. Allocation specifies the relative independence of one grammar to another grammar. In *independent* relations, the grammars remain fully formed with no direct connection to each other, thus operating in parallel. This is found in all the examples in Figure 4. In *substitutive* relations on the other hand, the units or combinatorics of one grammar function within and are determined by another grammar. This occurs in all the examples of the [_S_ Subject—Picture_Verb_–Object] construction in Figure 1, where the verb-function of the images is determined by the grammar of the verbal sentences.

Below, we further elaborate on the formalisms of these types of interactions. We will then elaborate on their manifestations in the contexts of unimodal and multimodal interactions.

### Symmetry

As described above, symmetry is a principle of grammatical interactions (GI, from here on) that characterizes the relative complexity of interacting grammars. As our broad classes involve simple and complex grammars, symmetry describes the ways these classes interact, as detailed in Table 2. Crossing simple and complex grammars gives rise to both symmetrical relations, maintaining the same complexity of grammars, while asymmetrical interactions arise when grammars of different complexity are used. We now turn to detailing the properties of these relations.

**Table 2:** Possibilities for multimodal interactions between grammars of two modalities.

		**Simple**			**Complex**		
		**One-unit**	**Two-unit**	**Linear**	**Simple phrase**	**Categorical**	**Recursive**
Simple	One-unit					
	Two-unit	Symmetrical Simple			Asymmetrical		
	Linear						
Complex	Simple phrase						
	Categorical	Asymmetrical			Symmetrical Complex		
	Recursive						

#### Symmetrical Simple

When multiple grammars are simple, we describe them as *Symmetrical Simple* interactions. We here collapse all of these simple types (i.e., one-unit, two-unit, and linear grammars) into a single formalism using optionality (notated with parentheses, and a Kleene star ^*^ for potential repetition) whereby two grammars are interacting in (GI-1):

(GI-1) Symmetrical Simple

GS_1_: [_Utterance_ Unit – (Unit^*^)]

GS_2_: [_Utterance_ Unit – (Unit^*^)]

Each of these schemas describes a simple utterance with at least one unit, possibly elaborated into two or a linear sequence. This type of interaction occurs when a single gesture comes with a single word (like making a deictic pointing gesture along with uttering “that”). It could also describe a single textual word along with a picture, such as a meme with an image and one word, or a single word along with an emoji (“Nice!
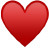
”).

#### Symmetrical Complex

Symmetrical relationships can also persist between complex grammars. We again collapse all three complex grammars (categorical grammars, simple-phrase grammars, and recursive grammars) into a single formalism that attempts to capture these complexities. As before, simple-phrase and recursive grammars require two interacting structures, which allows the embedding of the phrasal level schema into the utterance level schema. Thus, we here divide these parts by a comma, as in (GI-2):

(GI-2) Symmetrical Complex

GS_1_: [_Utterance_ Unit_x_/Phrase${}_{\text{x}}^{*}$], [_Phrase_ Unit_x_/Phrase_x_^*^]

GS_2_: [_Utterance_ Unit_x_/Phrase${}_{\text{x}}^{*}$], [_Phrase_ Unit_x_/Phrase_x_^*^]

Within each grammatical structure, both schemas specify that a unit or phrase, potentially of a particular category (subscript X), forms an utterance or a phrase. An example of such an expression that involves two complex grammars would be a comic strip, like in Figure 4C, with a complex visual narrative sequence interacting with sentences in text (such as in emergent carriers like balloons or captions). Another example of such an interaction might be the expression of a bimodal bilingual who both speaks and signs at the same time. In video, subtitles appearing while a person talks would also use a Symmetrical Complex interaction, with degrees of redundancy of the meaning between text and speech for whether it is the same language (depending on the quality of subtitling) or different languages (depending on the quality of translation).

#### Asymmetrical

Interactions between one simple grammar and one complex grammar are described as asymmetrical. Using the same formalisms as above, asymmetrical interactions are characterized as in GI-3:

(GI-3) Asymmetrical

GS_1_: [_Utterance_ Unit – (Unit^*^)]

GS_2_: [_Utterance_ Unit_x_/Phrase${}_{\text{x}}^{*}$], [_Phrase_ Unit_x_/Phrase${}_{\text{x}}^{*}$]

A typical example of an asymmetrical grammatical interaction is a gesticulation that runs concurrently with speech, often using a one-unit grammar along with a complex sentence grammar (McNeill, 1992; Clark, 1996). Similarly, a single emoji placed at the end of a typed sentence entertains the same relationship (Cohn et al., 2019; Gawne and McCulloch, 2019). These are the examples in Figures 4A,B, which both use asymmetrical interactions. Conversely, a visual sequence with onomatopoeia, such as a fight scene with sound effects (like a sequence of one person punching another with the text “Pow!”), would have a complex narrative grammar along with the one-unit word. All of these examples are asymmetrical in the interactions between their grammars.

### Allocation

While symmetry involves the relative complexity of the grammars involved, allocation relates to the way in which those grammars are distributed relative to each other. This distribution gives us two types: Independent and Substitutive. Independent allocation allows each grammar to exist on their own without any direct interaction, while Substitutive allocation places one grammar as a unit within another grammar. These notions have much in common with prior work such as Clark's (1996) description of “concurrent” and “component” co-speech gestures, here now elaborated across all modalities and operationalized to grammatical interactions specifically. In formal terms of our Parallel Architecture, allocation can be captured by how different grammars may be coindexed.

#### Independent

**Figure 6:**
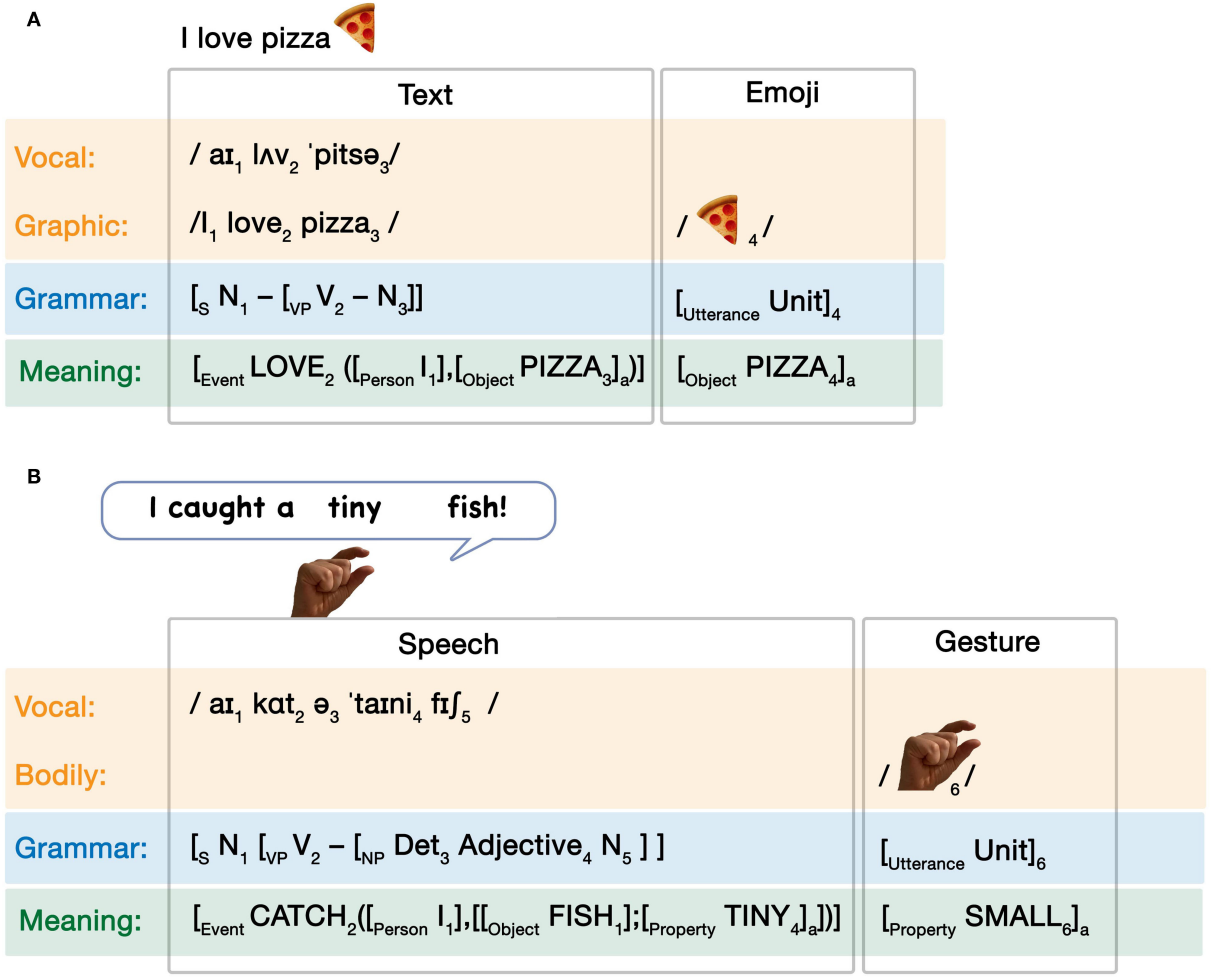
Independent grammatical interactions between **(A)** text and emoji and **(B)** speech and gesture.

We begin with Independent allocations. In this allocation, the units of both systems are independently distinguishable for whatever grammatical roles may be played (if any) within and across systems. Independent allocation occurs in all interactions in Figure 4. The critical insight here is that the grammatical allocation is mediated by the interactions between the modalities. That is, the temporal or spatial correspondence between modalities themselves allows for the interfacing of grammars, but on their own, the grammars remain independent. Allocation between grammars here is imposed by the circumstances of the modality interfaces. Consider an interaction between text and an emoji like: “I love pizza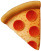,” formalized in Figure 6A.

Here, the pizza emoji is merely spatially integrated with the sentence (by following it). This spatial correspondence creates an interface between the adjacent word “pizza” and the emoji. By maintaining both the word “pizza” and the pizza emoji, it warrants a coreferential relationship, leading to coindexation in the conceptual structure (notated as subscript “a”). The grammars themselves (one-unit emoji and a textual sentence) remain independent. A similar interaction appears between the spoken sentence “I caught a tiny fish” with an accompanying pinching gesture to reinforce the small magnitude (Woodin et al., 2020), as in Figure 6B.

Because these grammars remain separate, so do their individual semantics. This independence is what invites coreference between the meaningful elements, broadly as balanced or imbalanced semantic weight, and more specifically as expressed by a range of coreferential connections. In the example above, overall this interaction creates an imbalanced relationship because the text carries more semantic weight than the gesture. This creates an “included” coreferential relationship where one word semantically overlaps with the gesture, but the rest of the verbal utterance is not reflected in the bodily modality. In independent allocation, the grammars work to package the meaning of each modality separately, creating the need for multimodal meaning to emerge outside the context of grammatical constraints. That is, multimodal meaning in this case arises at the level of conceptual structure alone, given the separate but interacting contributions of each expression.

In these allocations, both the modalities and the meanings work to create connections between messages, while the grammars only contribute to their own expressions but not to the overall multimodal message. To reiterate, in these cases the modalities interface to create sensory alignment and/or integration in temporal or spatial correspondence. The grammars of these modalities work to package the message of each expression independent from each other. This independence puts greater demands on the conceptual structure to integrate the meanings of those separate messages, requiring the search to establish coreference between the semantics of each modality and the inferences necessary to resolve such coreference.

#### Substitutive

While independent allocation keeps each grammar separate, substitutive allocation incorporates the grammars together into one sequence. Substitution is here defined as when the grammar used by one expression is inserted as a unit within another grammar. We refer to the inserted expression as the “substitution” and to the grammar that receives the substitution as the “matrix grammar.” Thus, the grammatical role of the substitution may be determined by the top-down sequencing schema of the matrix grammar. For example, in the sentence “I love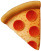,” the pizza-emoji is substituted for a noun in the matrix grammar, here as the Direct Object noun of a sentence. Unlike the heart in Figure 3C, which is entrenched in the lexicon with the grammatical role of verb, here the pizza emoji is itself not encoded in the lexicon with the grammatical role of a noun. This poses a problem for unification at the level of grammar, because the pizza emoji is not encoded as a noun—and cannot become one—that can fill a noun slot in the syntactic construction. For example, the pizza emoji cannot express case, like regular nouns.

**Figure 7:**
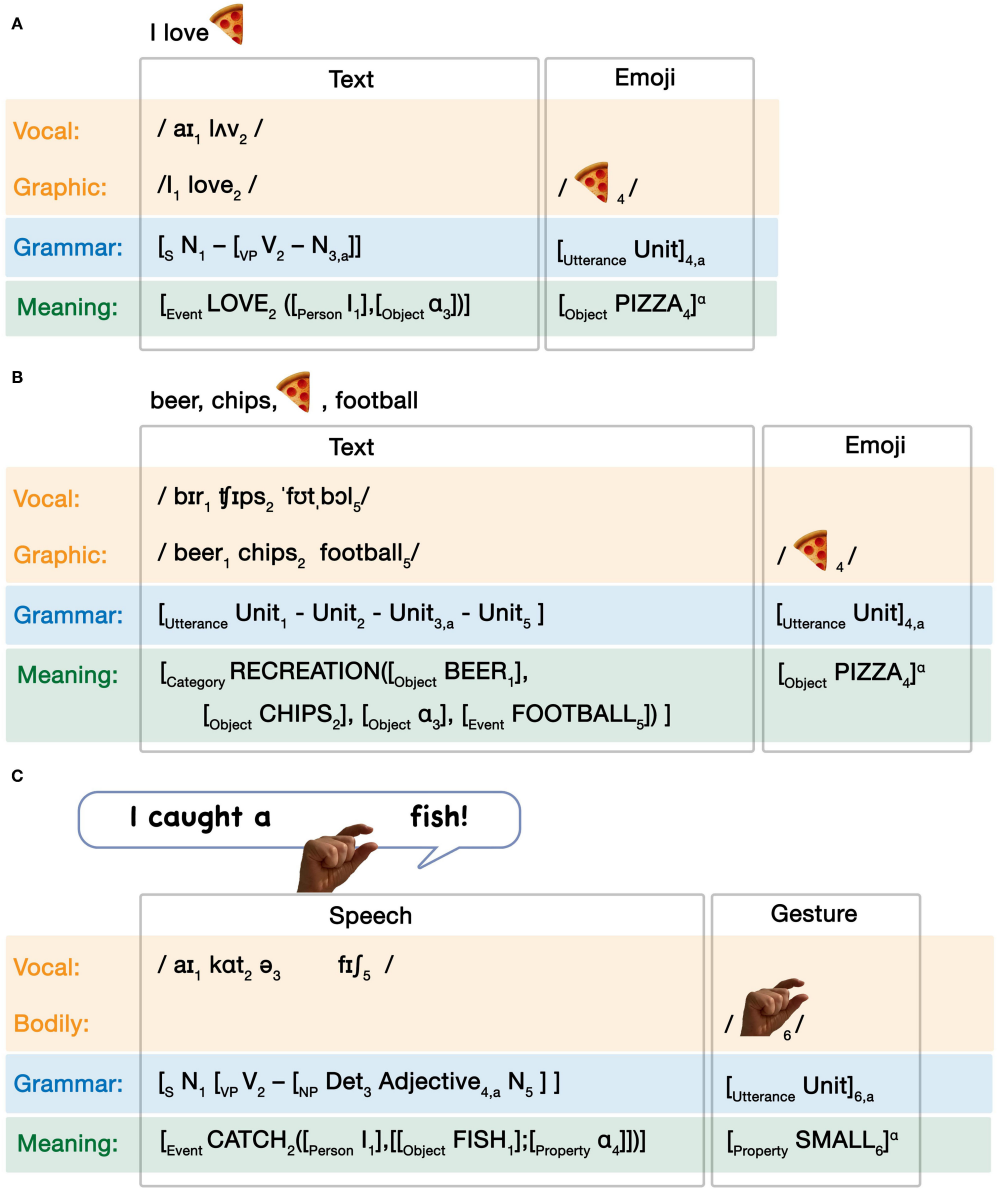
Substitutive grammatical interactions between **(A)** a sentence of text and emoji, **(B)** a list of text and an emoji, and **(C)** speech and gesture.

Below, we address this issue by assuming that the emoji's placement into a canonical sentence position following the transitive verb “love” allows it to fulfill both the semantic and grammatical argument structures. This is depicted in Figure 7A.

Here, the modalities have no explicit interface, because the expressions of each modality become units within a single sequence, rather than co-occurring expressions. In the grammatical structure, the pizza emoji appears as an unmarked unit, while the text invokes a canonical transitive sentence construction. Substitution is thus characterized formally by an index to the entire substituted message on the outside of the utterance (here, subscript “a”), which coindexes to a single unit within the matrix grammar (here, the direct object noun). In conceptual structure, the verb “love” licenses a transitive event with an argument structure specifying both an agent (here “I”) and a patient. As the patient is not expressed overtly in the text, this argument is fulfilled with a binding operator (α) which then links to the conceptual structure of the pizza emoji, such that it fulfills the semantic argument of the event structure. Overall then, the substitution results in one modality fulfilling a grammatical role within, and determined by, the grammar of another modality, thereby coalescing their meaning.

**Figure 8:**
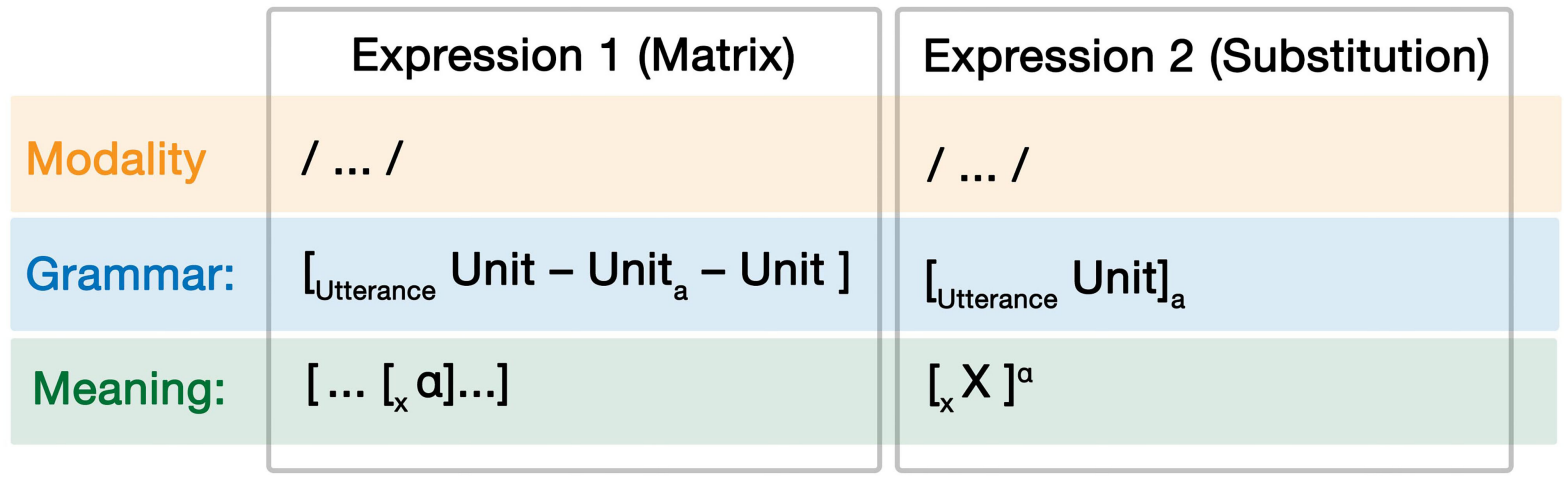
Abstracted correspondence schema for substitutive allocation.

Note that the formal challenge of substitution is how can representations of one type of expression (e.g., “unit”) unify with those of another (e.g., “noun”)? Our proposal is that unification occurs solely within the conceptual structure, such that a conceptual category corresponding to Expression 1 (like the Object of PIZZA of a pizza emoji) is licensed to be unified with a conceptual category corresponding to Expression 2 (like the Object slot made available by the transitive event LOVE). Through the prototypical correspondences of that unified conceptual structure, the substituted unit can thus play a role within the matrix grammar (i.e., the unified Object prototypically corresponds to a noun, which can satisfy the grammatical constraints of the transitive verb *love*). We articulate this as a generalized correspondence schema in Figure 8. To reiterate, the binding operator (α) reflects the unification of meaning of the substituted unit into the matrix expression's conceptual structure. This creates the possibility of the substituted unit's grammatical structure (whatever it may be) being inserted into a grammatical unit within the matrix grammar (coindex “a”).

Under this view, unification does not occur directly within the grammars, but is in a sense conceptually “coerced” within the grammar. To restate this more simply, the meaning of the substituted unit can satisfy the allowable meanings of the grammatical slot, and thereby can lead to acceptability of being a grammatical substitution. Note, however, that in some cases, this may lead to less satisfactory meaning unification. For example, if a substituted unit does not readily satisfy the constraints of the conceptual structure offered by the matrix grammar, this may lead to a less well-formed grammatical substitution. Consider substitutions like “I love
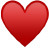
” or “I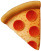 eating lunch” where, despite the shared semantic fields of the substituted elements with the matrix grammar, the substitutions appear less felicitous. Indeed, substitutions of emoji meanings that align with their grammatical context are readily integrated into the grammar, but substituted emoji with less appropriate meanings create downstream processing costs (Cohn et al., 2018; Scheffler et al., 2021).

Given the orthogonal relationships of grammatical symmetry, substitutions can thus vary across all types of symmetry. Substitutive allocation in the context of Symmetrical Simple interactions can be formalized as:

(GI-4) Symmetrical Simple substitutive allocation

GS_1_: [_Utterance_ Unit – (Unit^*^)]_1_

GS_2_: [_Utterance_ [Unit] – [Unit]_1_ – [Unit]^*^]

In this case, each grammatical structure is an utterance whereby the units have no prespecified categorical roles, and may be limited to one-unit, or to sequences using two-units or a linear grammar of an unlimited length (as suggested by the Kleene star ^*^). For a sequence to allow for units to be substituted, the matrix grammar in a Symmetrical Simple substitutive allocation needs to use a linear grammar (here GS_2_), while the substitution can vary across levels of simple complexity (GS_1_). For example, imagine a list where one of the items is fulfilled by an image rather than a word: “beer, chips,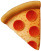, football.” We formalize this as in Figure 7B.

Again, the pizza emoji is integrated in the modality by virtue of its sequencing with the text, and it remains a single utterance with a conceptual structure consistent with the substitution in Figure 7A. This consistency reflects the integrity of the lexical entry of the pizza emoji as a unit. The textual list then uses a linear grammar whereby units lacking a grammatical category are ordered sequentially, and the whole utterance of the pizza emoji is coindexed to a single unit within that linear grammar (subscript “a”). The conceptual structure here just specifies a broader semantic field related to, say, recreation, where the semantics of the pizza emoji joins this broader category linked through the binding operator (α). Thus, in Symmetrical Simple substitutive allocation units from one expression can be inserted into a matrix grammar of another, but no further grammatical role is fulfilled because the linear grammar itself does not specify grammatical roles, such as the pizza emoji playing the role of a noun in Figure 7A.

Grammatical roles do become specified in Asymmetrical substitutive allocation. Here, the complex grammar of one expression uses grammatical roles, which the substitution using a simple grammar can inherit. This is what occurs in our example sentence “I love
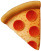
” in Figure 7A, where the pizza emoji acts as a noun in the textual sentence. Generalized, asymmetrical substitutive allocations can be formalized as in (GI-5):

(GI-5) Asymmetrical substitutive allocation

GS_1_: [_Utterance_ Unit – (Unit^*^)]_1_

GS_2_: [_Utterance/Phrase_ [Unit_x_/Phrase_x_] – [Unit_y_/Phrase_y_]_1_ –[Unit_z_/Phrase_z_]^*^]

Again, substitutions coindex the whole utterance of the substitution to a unit inside the utterance of the matrix grammar. While our formalized example in Figure 7A shows an image inserted into a textual sentence, multimodal substitutions of “component” (Clark, 1996) or “language-like” (Kendon, 1988; McNeill, 1992) gestures into the syntax of speech are also well attested (Kendon, 1988; McNeill, 1992; Clark, 1996; Fricke, 2013; Ladewig, 2020). For example, this would occur when speaking “I caught a <small pinching gesture> fish,” where the pinching hand gesture fulfills the role of a noun in the sentence corresponding to the notion of small magnitude (Woodin et al., 2020). We diagram this scenario in Figure 7C, which follows the same principles as our other substitutive examples in terms of co-indexation of grammars and alpha binding of conceptual structures. A reverse modality relationship occurs in bimodal bilinguals, who have proficiency in both a spoken language and sign language and have been observed to codeswitch (Emmorey et al., 2008). This codeswitching is a substitution of spoken words into the sign language grammar.

**Figure 9:**
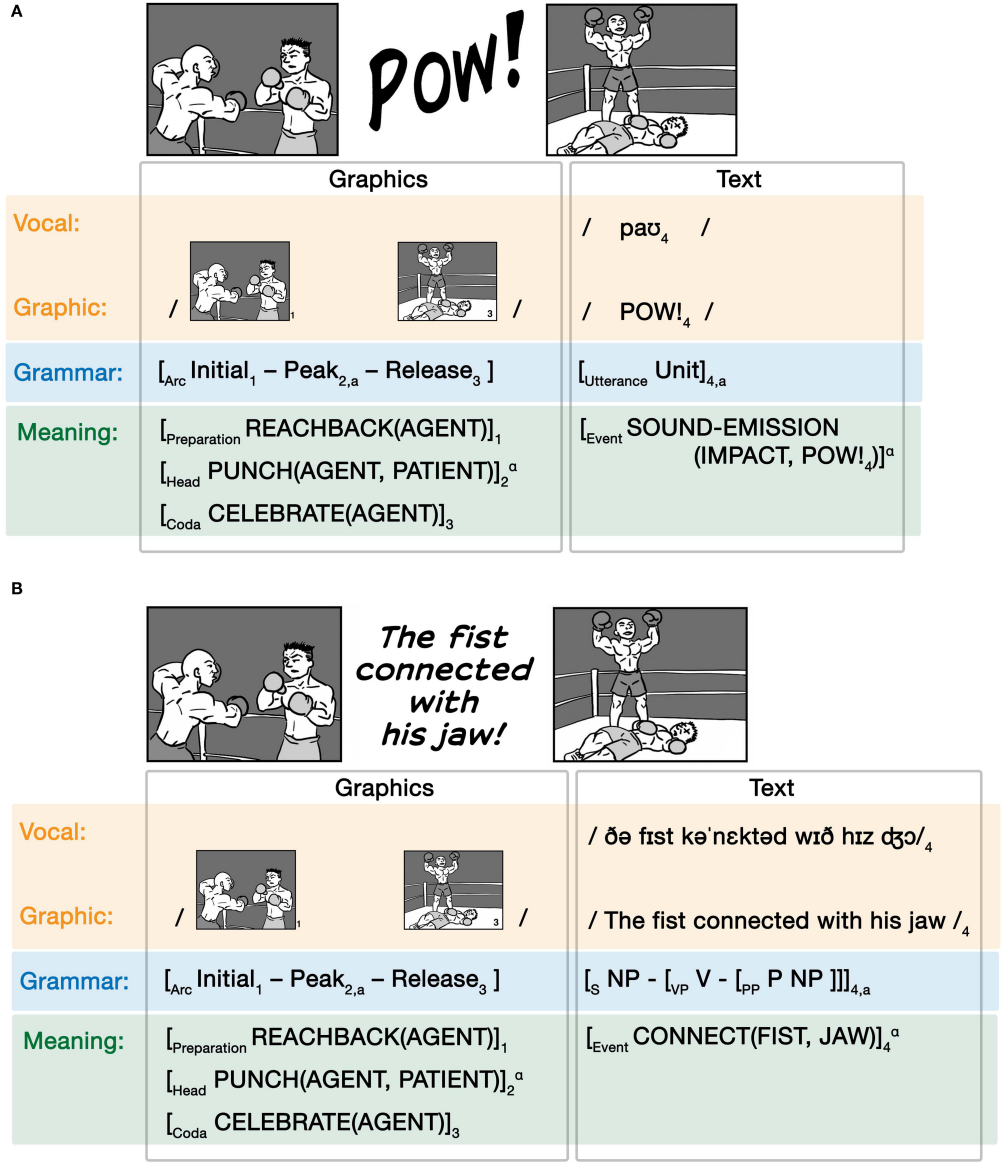
Substitutive grammatical interactions between a visual narrative sequence and **(A)** an onomatopoeia and **(B)** a sentence.

Units of text can also be inserted into a visual sequence that uses a complex grammar, such as in Figure 9A. Here, we first see one boxer reach back his arm while approaching another boxer, followed by the word “Pow” and then see a depiction of the first boxer standing over the second. We infer here that a punch occurred which must have knocked out the second boxer. However, we do not see this action: the climactic event of the visual narrative is replaced by an onomatopoeia, which sponsors inference of an event through the sound that it emits (Goldberg and Jackendoff, 2004; Jackendoff, 2010). We diagram this relationship in Figure 9A.

In this example, the onomatopoeia of POW! substitutes for the Peak, i.e., the climax, in the visual narrative sequence. The matrix grammar of the visual sequence depicts the Initial and Release states of the narrative, which correspond semantically to the agent boxer reaching back to punch and to the agent's subsequent celebration at the knock out. Here we simplify the conceptual structure to focus only on the agent's actions, but a complete notation would also include an independent articulation of the patient's event structure which would coindex to the agent's events. The description of the event structure through the Preparation-Head-Coda schema is a concise notation to describe discrete events (Jackendoff, 2007; Cohn et al., 2017). The graphic structure here leaves the punching event unseen, but it instead is implied through the corresponding onomatopoeia which represents a sound emitted by an impact (here, inferred as the impact of the punch), again linked through the binding operator (α). Substitutions of onomatopoeia for narrative Peaks are both a frequent type Asymmetrical substitutive allocation and an entrenched constructional pattern for sponsoring inferences in visual narratives (Cohn, 2019,^1^).

Relationships between complex grammars can also use substitutive allocation. In these cases, the substitution utterance has a complex grammar of its own such that its internal parts have their own grammatical roles, but the utterance as a whole plays a role as a unit in the matrix grammar of the other expression. Formalized, this appears as in (GI-6):

(GI-6) Symmetrical Complex substitutive allocation

GS_1_: [_Utterance/Phrase_ [Unit_x_/Phrase_x_] – [Unit_y_/Phrase_y_] – [Unit_z_/Phrase_z_]^*^]_1_

GS_2_: [_Utterance/Phrase_ [Unit_x_/Phrase_x_] – [Unit_y_/Phrase_y_]_1_ – [Unit_z_/Phrase_z_]^*^]

Again, this relationship is characterized by the whole substituted utterance coindexed with a unit in the matrix grammar. An example of such Symmetrical Complex substitutive allocation would occur when a whole sentence replaces the Peak in a visual narrative sequence, as in Figure 9B. Like in the Asymmetrical example in Figure 9A, this Symmetrical Complex substitution replaces the Peak event in a visual narrative for text, only here the text is a whole sentence rather than a single unit (Cohn, 2019; Huff et al., 2020). This substitution has its own complex grammar (here simplified in notation) that uses categorical roles and constituent structure, but as a whole plays a categorical narrative role determined by the matrix grammar. Again, this substitution is indicated by a coindex (“a”) of the sentence into the narrative structure, while the semantics are linked through the binding operator (α). We can also imagine the reverse situation, where an image sequence appears between words or clauses within a sentence.

Overall, in these substitutive allocations, the modalities thus are not interfaced in a temporal or spatial correspondence, but rather there is a sensory “switching” between modalities such that one expression concludes, a different modality begins and ends, and the original expression continues. As a result, no interfacing arises at the modality level, because no composite signals can be made out of integrating separate expressions into a holistic unit. In other terms, in independent allocations, the modality interfaces provide cues (synchronicity of speech and gesture, spatial proximity or connection for text and images, etc.) which give rise to unification operations within the conceptual structures. Unlike independent allocation, in substitutive allocation it is the grammar that works to integrate these multimodal messages by inserting an expression of one modality as a unit into the dominant matrix grammar of another modality. The result is that the grammar facilitates the access to and unification of meaning from both modalities. This precludes co-referentiality between the semantics of the modalities, thus giving rise to the need for a binding operator (α) to link the meanings. That is, because the modalities themselves remain separate, they do not contribute independent expressions needed to connect. Instead, the grammar facilitates this meaning, whether or not it invites conceptual integration.

Because grammars mediate the meaningful connections in substitutive allocation, our descriptions of balanced and imbalanced semantic weight no longer apply. While in (im)balanced relationships, meaning is negotiated through how modalities establish coreference to each other; in a substitutive relationship, the modalities each contribute independent semantics, and no units explicitly coreference each other. Therefore, we need to introduce an additional semantic interaction that is characterized solely by the substitutive allocation. This we call a ***compositional*** multimodal meaning, where semantic interactions arise from the unification of meaning facilitated by the insertion of the meaning of one modality into the grammatical structure—and thus the corresponding conceptual structure—of another modality, while the problem of absent coreference persists despite the grammatical substitution.

These types of interactions characterize the relationships between grammars alone, in the abstract. Though we emphasize that substitutive allocation may occur through grammars in multimodal interactions, all allocations also occur *within* modalities. In the vocal modality, ideophones are a lexical class of typically one-unit words that are prevalent in many of the worlds languages (Dingemanse, 2017). These expressions show morphosyntactic independence—often placed at the end of sentences—yet they can also be inserted into sentences to take on grammatical roles (Dingemanse, 2017). In the bodily modality, similar asymmetrical allocation occurs with gestures that accompany grammatical sign language (Marschark, 1994; Emmorey, 2001).

Unimodal substitution also arises in interactions between different representational systems, such as in codeswitching between two languages—i.e., where the units, of varying sizes, of one language are inserted into the matrix grammar of another language (Kootstra, 2015; Muysken, 2020). Like multimodal substitutions, “insertional” codeswitching is motivated by cognates for substituted words or clauses between languages, and in many cases the morphosyntax of the utterance comes from only the matrix grammar (Myers-Scotton, 1997, 2002). As a result, codeswitches are more often content words (like nouns) than function words. Multimodal substitutions of emoji into sentences are consistent with this, as people more often replace pictures for certain grammatical categories (nouns, adjectives) in sentences over others (verbs, adverbs) (Cohn et al., 2019). We might think of this “unimodal switching” between languages as a type of substitution, whereby the units come from different representational systems within the same vocal modality, rather than a “multimodal codeswitching” of substitution from different modalities. This aligns with the idea that a broader lexicon distinguishes lexical items with features for different languages (Jackendoff and Audring, 2020), which here thus would extend to a lexicon across and between modalities. Thus, again allocation characterizes the interactions between grammars, no matter the modality or representational origins of those grammars.

It is worth noting that psycholinguistic research supports the idea that substituted elements from one modality readily integrate into the grammar and meaning of the matrix modality, despite differences in processing the modalities themselves. For example, reading times for grammatically congruous substituted emoji were slower compared to words in sentences, but viewing times for grammatically incongruous or homophonous rebus emoji were even slower (Cohn et al., 2018; Scheffler et al., 2021). In addition, viewing times for sentences substituted for images in visual sequences (symmetrical substitution, as in Figure 9B), were also found to be slower than their substituted pictures (Huff et al., 2020). However, onomatopoeia in visual narratives were actually viewed faster than the pictures they substituted^1^. Thus, while some work suggests that switching modalities may incur costs, this may either be due to the front-end change in type of representation (graphics to text, or vice versa), or may simply be a matter of relative complexity in the visual representation.

The integration of substitution and matrix grammar is further suggested by studies of grammatical and semantic processing. Pictures inserted into sentences are comprehended with accuracy comparable to all-verbal sentences (Potter et al., 1986; Cohn et al., 2018). Substituted emoji within sentences that better maintain the expectations of the written grammar incur no sustained costs (ex. *John loves eating*
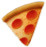
…), while “ungrammatical” pictures (ex. *John*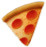 *eating pizza…*) create spillover costs that persist after the substitution (Cohn et al., 2018). Substituted emoji are also viewed faster than independent allocations of emoji placed at the end of sentences (Cohn et al., 2018). Finally, neural responses indexing semantic processing (the N400, as measured by event-related potentials) are modulated by congruity or predictability of a substitution with the content of its matrix sequence, whether for images substituted into text (Nigam et al., 1992; Ganis et al., 1996; Federmeier and Kutas, 2001,^2^) or for text substituted into a visual narrative sequence (Manfredi et al., 2017). Together, these findings imply that, while modalities themselves may incur front-end costs, substituted elements readily integrate with their matrix modality both across semantics and grammar.

## Conclusion

We have presented an expansion of Jackendoff's Parallel Architecture which accounts for both unimodal and multimodal expressions as emergent interactions within a holistic system with primary structures of modality (vocal, bodily, graphic), grammatical structures, and conceptual structures. As this model allows for both unimodal and multimodal expressions, interactions within each of its structures allow for a wide range of variation in expressions. We have primarily focused here on grammatical interactions, arriving at two dimensions of variability. Symmetry characterizes the overall relative complexity of contributing grammars, while allocation describes the ways that those grammars are distributed.

It should be noted that allocation in many ways drives the overall multimodal interactions. When grammars remain separated in independent allocations, it entails that the modalities themselves remain separate. In these cases, modalities interface on their own (temporal or spatial correspondence) and require coreference across signals. By integrating grammars in substitutive allocation, the multimodal messages themselves become integrated. Thus, while the overt experience of multimodality occurs in the perception of the sensory modalities, and the understanding of their integration results through the conceptual structure, we may remain unaware of the covert interactions of the grammars that largely characterize how multimodal messages arise.

In addition, the ability for grammatical structures to substitute into each other has implications for the characteristics of the wider faculty of language. As substitution appears to apply unimodally within a language (ideophones, signers gesturing), unimodally across languages (codeswitching), and in multimodal interactions (text/image, speech/gesture), it implies that substitution does not merely occur between modalities, but between *grammars*, no matter their representational origin. Furthermore, given that substitution serves as a diagnostic more broadly for a linguistic test of complementary distribution, substitutive allocation can be taken as a defining distributional trait for inclusion into a broader linguistic faculty. One can substitute across expressive systems ***because*** these systems share their cognitive architecture. We claim the Parallel Architecture accommodates this evidence of cross-modal substitution. Thus, substitution can be used as a diagnostic for testing the degree to which modalities or representational systems overlap within a broader linguistic faculty.

Altogether, we have argued for two fundamental observations about human language and multimodality. First, expressions across modalities are not indivisible, but rather are decomposable into similar substructures which have classifiable interactions. Second, in order to accurately account for the structure and cognition of human language, multimodality must be addressed. We contend that any accurate accounting for language and multimodality must address these issues. That is, to address multimodality, but not its decomposable interactions, does not do justice to the complexity of multimodal expressions. At the same time, unimodal linguistic models fail to characterize the full and accurate complexity of human language. As we have argued, the Parallel Architecture provides a model of human expressive capacities capable of accounting for the richness demanded of the natural competence for multimodality.

## Data Availability Statement

The original contributions presented in the study are included in the article/supplementary material, further inquiries can be directed to the corresponding author/s.

## Author Contributions

NC and JS contributed equally to the theorizing and creation of this paper. All authors contributed to the article and approved the submitted version.

## Funding

Funding for open access publication fees is provided from Tilburg University.

## Conflict of Interest

The authors declare that the research was conducted in the absence of any commercial or financial relationships that could be construed as a potential conflict of interest.

## Publisher's Note

All claims expressed in this article are solely those of the authors and do not necessarily represent those of their affiliated organizations, or those of the publisher, the editors and the reviewers. Any product that may be evaluated in this article, or claim that may be made by its manufacturer, is not guaranteed or endorsed by the publisher.

## Footnotes

^1^Klomberg, B., and Cohn, N. (under review). *Picture Perfect Peaks: Comprehension of Inferential Techniques in Visual Narratives*.

^2^Weissman, B., Cohn, N., and Tanner, D. (in preparation). *Predictable Words and Emoji Neural Correlates of Verbal and Pictorial Lexical Prediction in Sentence Processing*.
